# Inhibitory effects of heat shock protein 90 blockade on proinflammatory human Th1 and Th17 cell subpopulations

**DOI:** 10.1186/1476-9255-11-10

**Published:** 2014-04-02

**Authors:** Stefan Tukaj, Detlef Zillikens, Michael Kasperkiewicz

**Affiliations:** 1 Department of Dermatology, University of Lübeck, Ratzeburger Allee 160, 23538 Lübeck, Germany

**Keywords:** 17-DMAG, Heat shock protein, T cell

## Abstract

**Background:**

Heat shock protein 90 (Hsp90), a chaperone that regulates activity of many client proteins responsible for cellular growth, differentiation, and apoptosis, has been proposed as an important clinical and preclinical therapeutic target in a number of malignancies and autoimmune diseases, respectively. In this study, we evaluated the effects of pharmacological Hsp90 inhibition on human proinflammatory T cell responses.

**Findings:**

Using anti-CD3 antibody-stimulated human peripheral blood mononuclear cell cultures, we observed that Hsp90 inhibition by non-toxic concentrations of the geldanamycin derivative 17-DMAG significantly blocked T cell proliferation, reduced IFN-γ and IL-17 expression on CD4^+^ T lymphocytes, and arrested secretion of proinflammatory IFN-γ, TNF-α, and IL-17, cytokines characteristic of Th1 and Th17 cells, respectively. These effects were associated with inhibition of NF-kB activity, upregulation of Hsp70 protein expression, and disruption of T cell-specific nonreceptor tyrosine kinase Lck activation.

**Conclusions:**

Our results further support the potential use of Hsp90 inhibitors in patients with autoimmune diseases where uncontrolled Th1 or Th17 activation frequently occurs.

## Introduction

Heat shock protein 90 (Hsp90) is an ATP-dependent molecular chaperone that is exploited by malignant cells to support activated oncoproteins, including many cancer-associated kinases and transcription factors, but has been also shown to exert potent immunomodulatory actions [1,2].

Several pieces of evidence show that Th1 and Th17 cells are pathophysiologically associated with several autoimmune diseases and that Hsp90 activity is required for IFN-γ and IL-17 signaling in these cell types, respectively [3-5]. Based on these findings and the observations that Hsp90 plays important roles in antigen presentation, activation of lymphocytes, macrophages, and dendritic cells [2], we and others claim that pharmacological inhibition of Hsp90 is a promising therapy to ameliorate inflammatory cascades in autoimmune diseases [6-11].

In the present study, we show that blockade of Hsp90 by the geldanamycin derivative 17-DMAG leads to inhibition of T cell proliferation, suppression of IFN-γ and IL-17 expression on CD4^+^ T cells, and attenuation of secretion of proinflammatory IFN-γ, TNF-α, and IL-17, cytokines characteristic of Th1 and Th17 cells, respectively.

## Methods

### Cell culture

Peripheral blood mononuclear cells (PBMCs) were isolated from venous blood of 11 healthy volunteers (age 25-35 years, mean 29.72 ± 3.47, 6 females and 5 males) by Ficoll-Paque (GE Healthcare Bio-Sciences) gradient centrifugation and cultured as described previously with minor modification [12]. Briefly, PBMCs were washed twice in PBS and resuspended at 0.5 × 10^5^ or 1 × 10^6^ cells per ml of medium (RPMI 1640 supplemented with 10% fetal calf serum, 2 mM L-glutamine, and penicillin/streptomycin). Cells were cultured in the presence of 1 μg/ml immobilized anti-CD3 mAb (BD Bioscience) in 24-well or 96-well culture plates in 5% CO_2_ at 37°C without or with different concentration of 17-DMAG (InVitrogen) for 24 hours, 72 hours, or 7 days.

The collection of blood samples was approved by the Ethics Committee of the University of Lübeck, and informed consent was obtained according to the Declaration of Helsinki.

### LDH cytotoxicity assay

Cytotoxicity of 17-DMAG was measured by a lactate dehydrogenase (LDH)-releasing assay using a Cytotoxicity Detection Kit (Roche) according to the manufacturer’s protocol and quantified using an ELISA plate reader. Briefly, PBMCs (1 × 10^6^ per ml) were incubated with 17-DMAG at different concentrations (0.1, 1, and 2.5 μM) for 24 hours. Cell lysis was determined by measuring the amount of LDH released into the culture medium.

### Proliferation assay

A total of 0.5 × 10^5^ PBMCs were cultured in the presence of plate-bound anti-CD3 mAb in 96-well plates. Stimulated cells were cultured alone or with different concentrations of 17-DMAG (0.1, 1, or 2.5 μM). After 6 days, cells were pulsed with BrdU for further 24 hours. T cell proliferation was measured using a colorimetric cell proliferation BrdU ELISA (Roche).

### Flow cytometric immunophenotyping

For analysis of intracellular cytokines, PBMCs were stimulated with anti-CD3 mAb without or with 17-DMAG (0.1 μM) for 72 h hours. A GolgiStop™ Protein Transport Inhibitor (BD Pharmingen™) was added together with phorbol-12-myristate-13-acetate (PMA) (50 ng/ml) and ionomycin (1 μg/ml) (Sigma) for the last 5 hours. Cells were washed, fixed, permeabilized, and stained for detection of intracellular cytokines using the following anti-human mAbs: anti-IFN-γ-FITC, anti-IL-17-PE, and anti-CD4-PERCP-CY5.5 (BD Pharmingen). Samples were analyzed using FACSCalibur flow cytometer (BD).

### Cytokine measurement

Supernatants of PBMC cultures were collected after a 72-hour treatment period with anti-CD3 mAb in absence or presence of 17-DMAG (0.1 μM). Supernatants were taken from cultures non-treated by GolgiStop, PMA, or ionomycin. Twelve cytokines (IL-2, IL-4, IL-5, IL-6, IL-10, IL-12, IL-13, IL-17A, IFN-γ, TNF-α, G-CSF, and TGF-β1) were measured by a Human Th1/Th2/Th17 Cytokines Multi-Analyte ELISArray Kit (Qiagen Biosciences).

### Determination of NFκB p65 activity

For analysis of NFκB p65 activity, PBMCs were stimulated with anti-CD3 mAb without or with different concentrations of 17-DMAG (0.1, 1, and 2.5 μM) for 24 hours. NFκB p65 activity was measured in cell lysates by a NFκB p65 ELISA kit following the manufacturer’s instruction (Enzo).

### Immunoblotting

Equal amounts of PBMC lysates were separated by 10% SDS-PAGE gel and transferred onto nitrocellulose membrane. The membrane was blocked with 3% milk in PBS for 2 hours, followed by incubation with rabbit polyclonal antibodies against NFκB p65 (1:1000; Santa Cruz Biotechnology, Inc.), Hsp70 (1:100; Stressgen), p-Lck (Tyr^394^) (1:1000; Sigma), and β-actin (1:1000; Cell Signaling Technology) at room temperature for 2 hours. Horseradish peroxidase-conjugated gout anti-rabbit antibodies (1:1000; Dako) were used as secondary antibodies. The bands were visualized using Amersham ECL Plus Western Blotting Detection Reagents (GE Healthcare). Relative NFκB p65, Hsp70, and p-Lck (Tyr^394^) protein concentrations were measured by densitometry.

### Statistical analysis

Data was analyzed by Student’s t-test or one-way analysis of variance (ANOVA) using Graphpad prism 5 (San Diego, California). A *P*-value <0.05 was considered to indicate a statistically significant difference.

## Results

### 17-DMAG arrests proliferation of T cells

**Figure 1:**
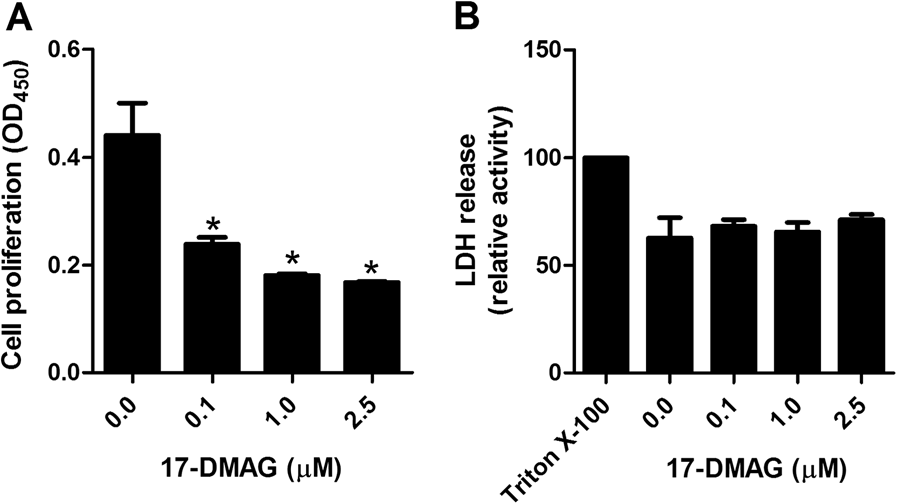
**17-DMAG inhibits proliferation of T cells. A** Proliferation response of human PBMCs stimulated by plate-bound anti-CD3 antibody (1 μg/ml) in absence and presence of 17-DMAG. Cell cultures were exposed to different concentrations of 17-DMAG and proliferation was assayed by ELISA after BrdU incorporation on the 6th day of treatment followed by 24 hours incubation. **B** LDH-based cytotoxicity ELISA measurement in culture medium after 24-hour incubation of PBMCs with different concentrations of 17-DMAG. LDH release from cells lysed with 1% Triton X-100 was regarded as 100%. The results are presented as mean values ± SEM of n = 4 healthy blood donors. *P < 0.05.

To determine whether 17-DMAG affects T cell proliferation, we isolated human PBMCs from healthy volunteers and stimulated them with anti-CD3 antibody in the absence or presence of different amounts of 17-DMAG. Cell proliferation was assayed by BrdU ELISA. T cell proliferation was inhibited by 17-DMAG in a dose-dependent manner (Figure 1A) and this inhibitory effect was observed upon non-toxic concentrations of the inhibitor (Figure 1B).

### 17-DMAG inhibits proinflammatory Th1 and Th17 cell subpopulations

**Figure 2:**
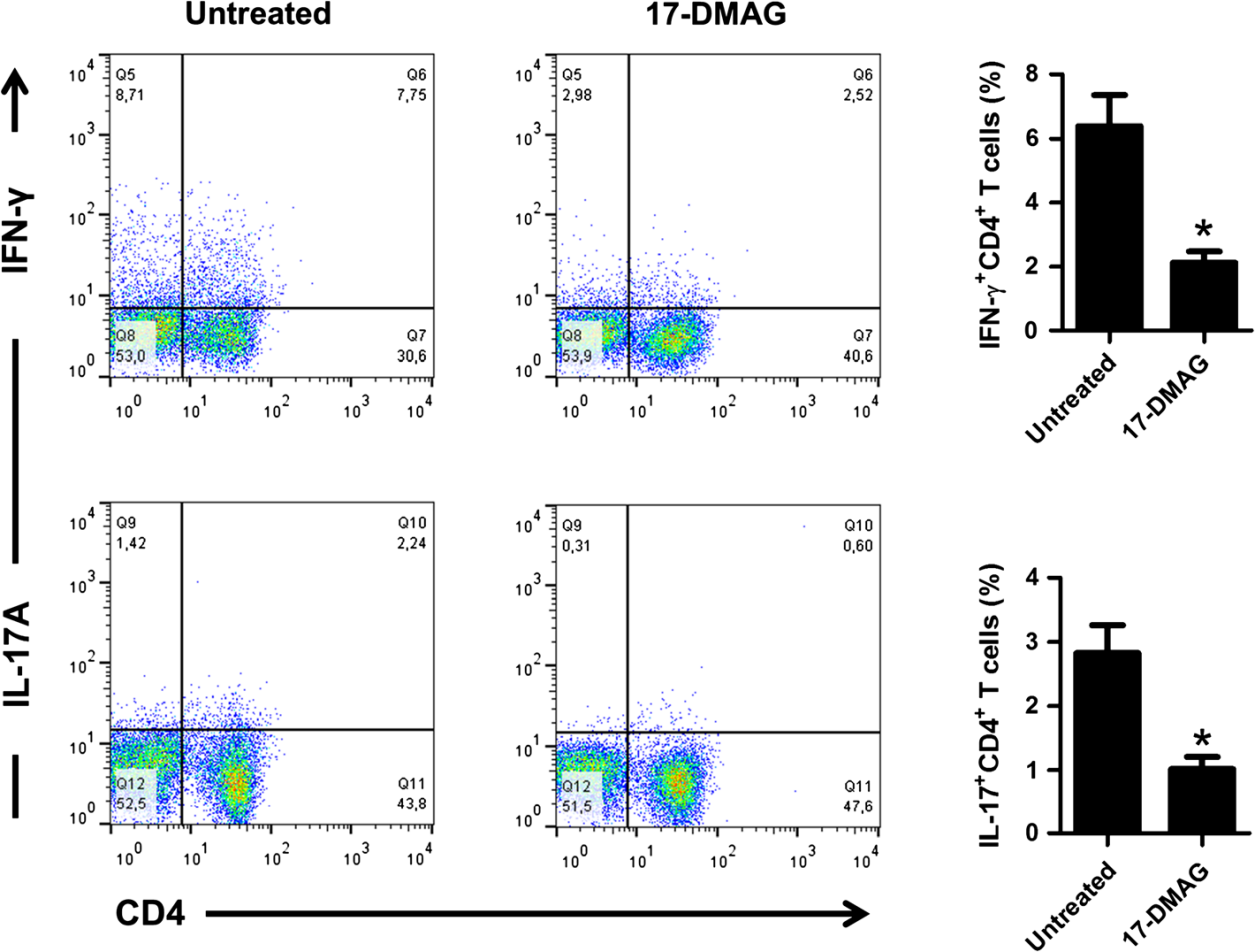
**17-DMAG reduces frequencies of proinflammatory Th1 and Th17 cell subpopulations.** PBMCs were stimulated with 1 μg/ml of plate-bound anti-CD3 antibody in absence or presence of 17-DMAG (0.1 μM) for 72 hours. GolgiStop™, PMA, and ionomycin were added in the last 5 hours of culture before permeabilization. Cells were washed, stained with anti-CD4, anti-IFN-γ, and anti-IL-17 antibodies, and analyzed by flow cytometry. The numbers in upper right quadrants of the representative dot blots show the percentage of positive cells of each CD4^+^ T cell subpopulation. The results are presented as mean values ± SEM of n = 4 healthy blood donors. *P < 0.05.

Next, we investigated the effects of 17-DMAG on proinflammatory T cell subpopulations [CD4^+^IFN-γ^+^ (Th1) and CD4^+^IL-17^+^ (Th17)] using flow cytometry. We observed that 17-DMAG significantly inhibited intracellular expression of IFN-γ and IL-17A in anti-CD3 antibody-treated PBMCs, representing Th1 and Th17 cell fractions, respectively (Figure 2).

### 17-DMAG dampens the secretion of proinflammatory cytokines

**Figure 3:**
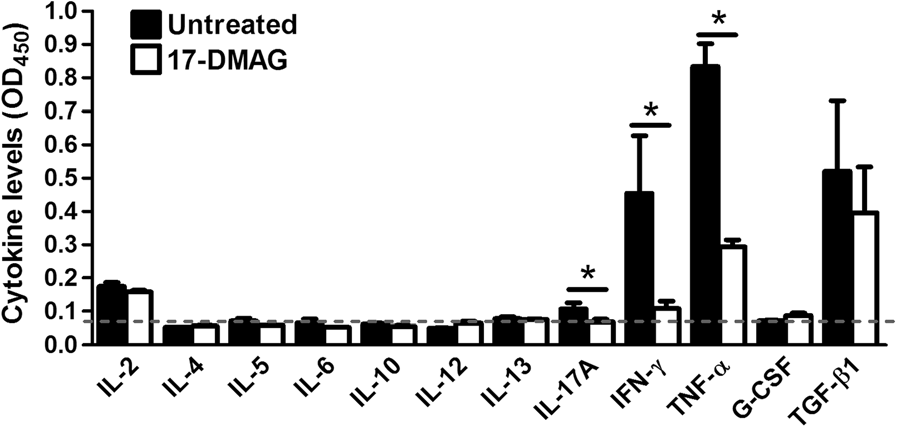
**17-DMAG arrests secretion of proinflammatory cytokines.** ELISA-based evaluation of the cytokines IL-2, IL-4, IL-5, IL-6, IL-10, IL-12, IL-13, IL-17A, IFN-γ, TNF-α, G-CSF, and TGF-β1 secreted into the culture medium by PBMCs which were stimulated with 1 μg/ml plate-bound anti-CD3 antibody and treated without or with 17-DMAG (0.1 μM) for 72 hours. The results are presented as mean values ± SEM of n = 3 healthy blood donors. The dotted line represents the mean detection limit of the assay. *P < 0.05.

Using ELISA, we further measured the effects of 17-DMAG on secretion of a broad array of PBMC-derived cytokines (IL-2, IL-4, IL-5, IL-6, IL-10, IL-12, IL-13, IL-17A, IFN-γ, TNF-α, G-CSF, and TGF-β1). The cytokines were assayed in culture medium of PBMCs stimulated with anti-CD3 antibody. The presence of 17-DMAG significantly inhibited the secretion of the proinflammatory cytokines IFN-γ (4.2-fold), TNF-α (2.8-fold), and IL-17A (1.5-fold). We found no significant influence of 17-DMAG on IL-2, G-CSF, and TGF-β1 secretion. The remaining cytokines were below the detection limit of the assay (Figure 3).

### 17-DMAG suppresses NFκB p65 activity

**Figure 4:**
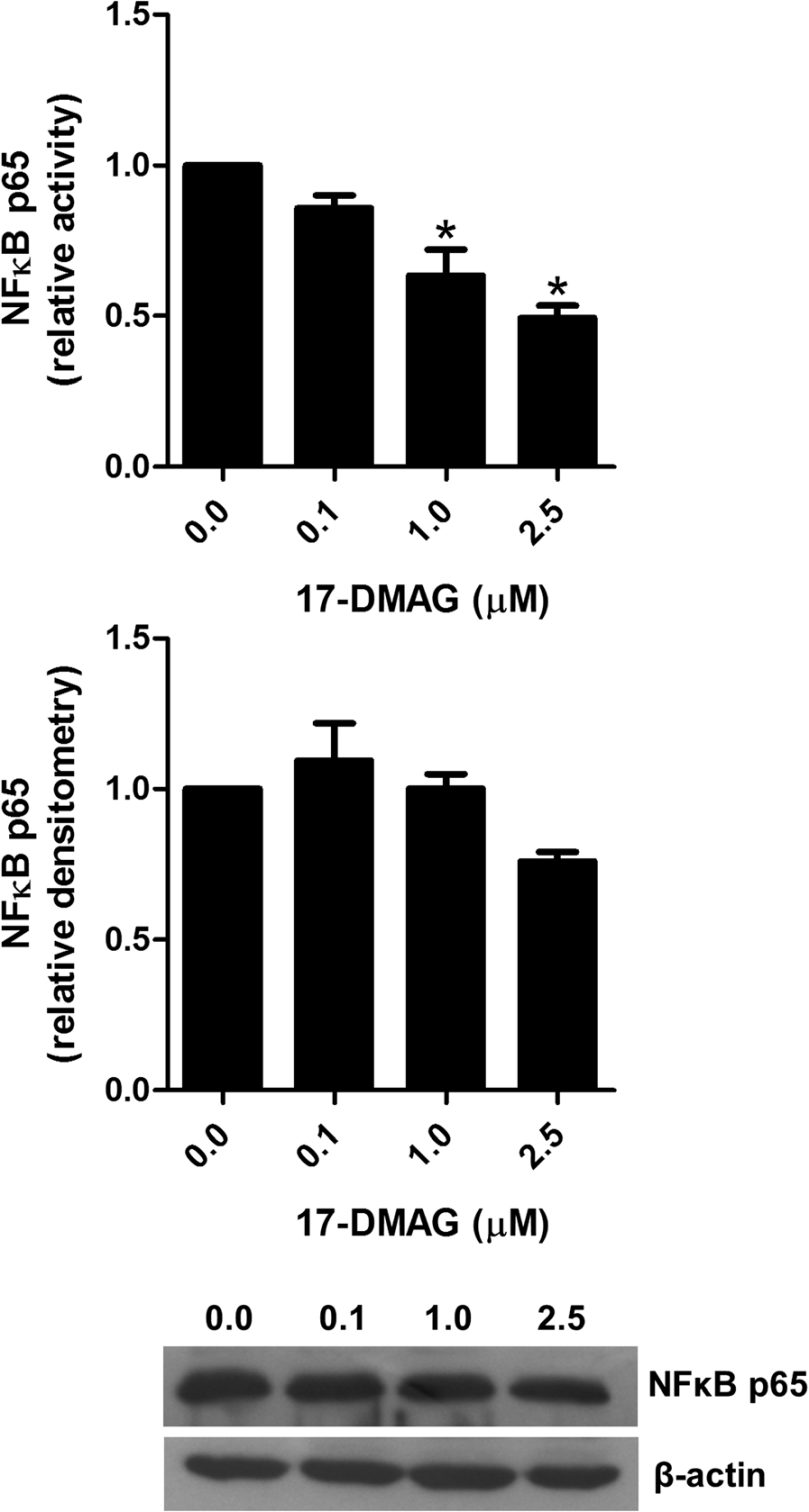
**17-DMAG blunts NF**κ**B p65 activity.** Analysis of NFκB p65 in PBMCs stimulated with 1 μg/ml plate-bound anti-CD3 antibody in absence and presence of different concentrations of 17-DMAG for 24 hours. NFκB p65 activity and protein expression was analyzed in lysates of these cell cultures by ELISA and immunoblotting, respectively. Protein concentration was expressed relative to the β-actin level using densitometry measurements. The results are presented as mean values ± SEM of n = 3 healthy blood donors. *P < 0.05.

To test whether Hsp90 inhibition had an impact on NFκB p65*,* its activity as well as protein expression was measured in cell lysates of anti-CD3 antibody-stimulated PBMCs by ELISA and immunoblotting, respectively. Our results revealed that the addition of 17-DMAG dose-dependently suppressed NFκB p65 activity without affecting its protein level (Figure 4).

### 17-DMAG upregulates Hsp70 expression

**Figure 5:**
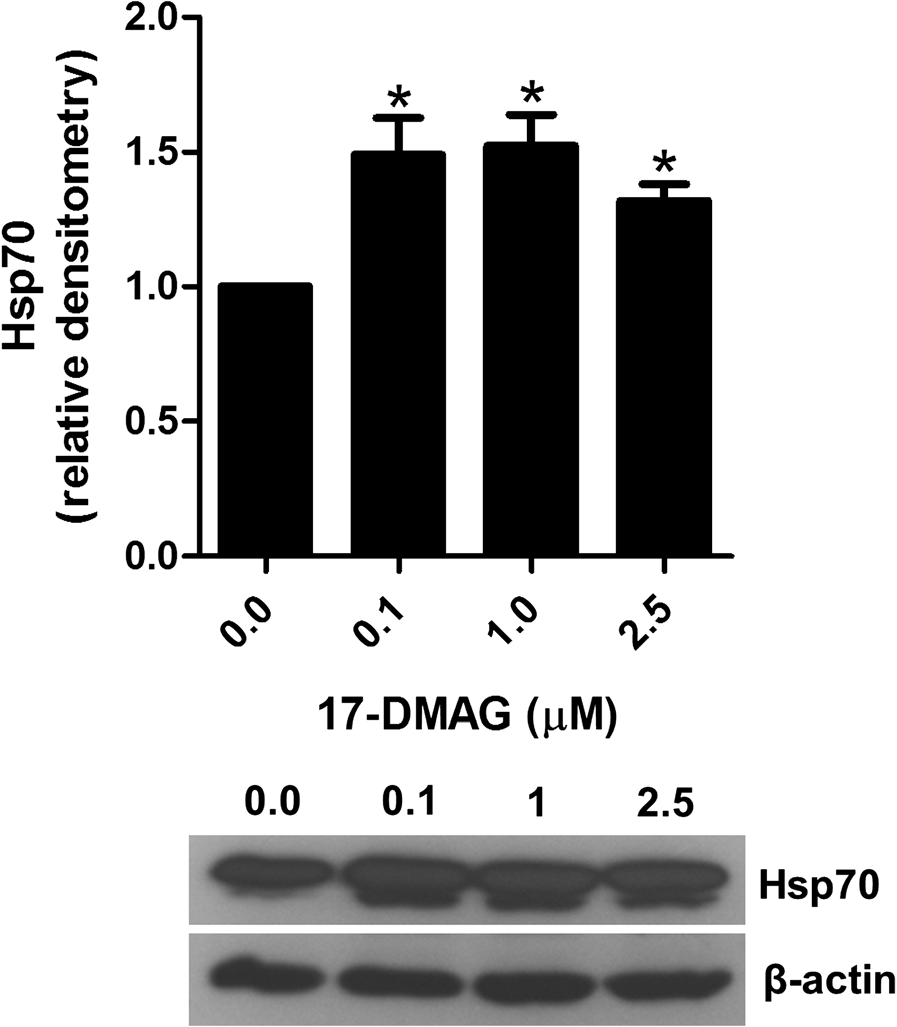
**17-DMAG induces Hsp70 expression.** Analysis of Hsp70 in PBMCs stimulated with 1 μg/ml plate-bound anti-CD3 antibody in absence and presence of different concentrations of 17-DMAG for 24 hours. Hsp70 protein expression was analyzed in cell lysates of these cell cultures by immunoblotting. Protein concentration was expressed relative to the β-actin level using densitometry measurements. The results are presented as mean values ± SEM of n = 3 healthy blood donors. *P < 0.05.

To investigate whether 17-DMAG influenced the expression of Hsp70, a common marker of Hsp90 inhibition, immunoblot analysis of lysates from anti-CD3 antibody-stimulated PBMCs was performed. Indeed, the addition of 17-DMAG to these cell cultures resulted in induction of Hsp70 protein expression (Figure 5).

### 17-DMAG blocks Lck phosphorylation

**Figure 6:**
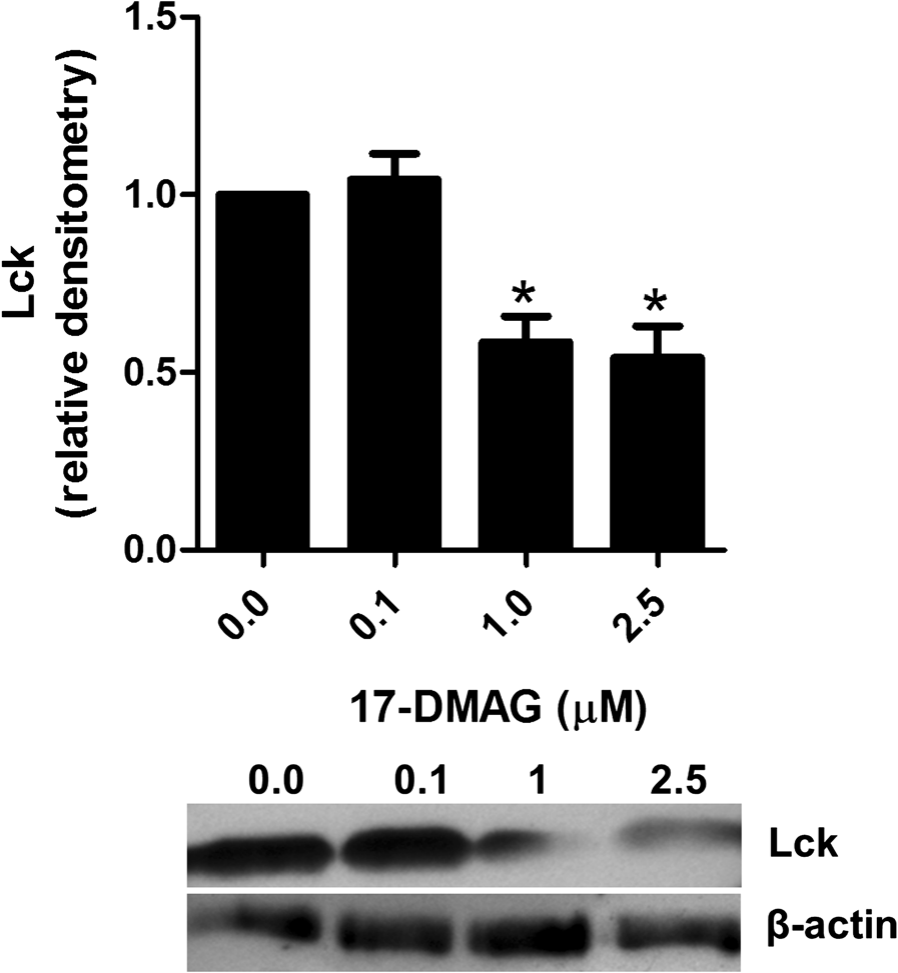
**17-DMAG disrupts Lck activation.** Analysis of Lck in PBMCs stimulated with 1 μg/ml plate-bound anti-CD3 antibody in absence and presence of different concentrations of 17-DMAG for 24 hours. Lck phosphorylation at Tyr^394^ position was analyzed in lysates of these cell cultures by immunoblotting. Protein concentration was expressed relative to the β-actin level using densitometry measurements. The results are presented as mean values ± SEM of n = 3 healthy blood donors. *P < 0.05.

To examine whether Hsp90 inhibition had an impact on T cell-specific nonreceptor tyrosine kinase Lck*,* its phosphorylation status was measured in cell lysates of anti-CD3 antibody-stimulated PBMCs by immunoblotting. We demonstrated that the addition of 17-DMAG dose-dependently suppressed Lck activation (Figure 6).

## Discussion

Here, we provide evidence that 17-DMAG, upon non-toxic concentrations, inhibited T cell proliferation and reduced percentages of Th1 and Th17 cells, which was associated with dampened Th1 (IFN-γ and TNF-α) and Th17 (IL-17) cytokine secretion. These results are in good agreement with previous studies reporting the capacity of Hsp90 blockers to inhibit proliferation of T lymphocytes ex vivo and to downregulate these proinflammatory T cell subtypes [4-11,13].

Since Th1 and Th17 cells are essential to the development of various autoimmune diseases, treatment strategies which aim at blocking of uncontrolled activation of such effector cell populations are highly warranted [3]. In fact, pharmacological blockade of Hsp90 has been reported to be an effective treatment in rodent models of T cell-mediated autoimmune diseases, such as autoimmune encephalomyelitis [6], rheumatoid arthritis [7,8], and systemic lupus erythematosus [9,10]. In addition, our research group recently demonstrated that, by downregulating T cell responses, treatment with Hsp90 inhibitors is also effective in mice with the experimentally induced autoimmune bullous disease epidermolysis bullosa acquisita [11].

Although the main focus of our experiments was to study the impact of 17-DMAG on Th1 and Th17 subpopulations, we cannot rule out but also not support that 17-DMAG additionally exhibited suppressive activity on other T cell populations such as Th2 and regulatory T cells since Th2 cytokines released from anti-CD3 antibody-stimulated PBMCs were below the detection limit of our assay and secreted IL-10 and TGF-β1, cytokines associated with regulatory T cell function, were also undetectable or not significantly inhibited in our study, respectively. In this context, it is worth noting that there is evidence in the recent literature that Hsp90 inhibition can promote rather than inhibit regulatory T cells, further supporting an antiinflammatory mechanism of action of Hsp90 blockers in terms of T cell responses [14,15].

Our current experiments further revealed that inhibition of T cells by 17-DMAG was associated with deactivation of NFκB and upregulation of Hsp70. While NFκB is a client of Hsp90 and one of the major transcription factors responsible for proliferation of T cells and their proinflammatory IFN-γ and IL-17 expression [16,17], Hsp70 is generally considered as a marker for effective Hsp90 inhibition and also regarded as potent antiinflammatory chaperone capable of inhibiting NFκB signaling pathways [18-20].

Corticosteroids, which are widely used to treat patients with autoimmune diseases, mediate their immunosuppressive effects through cytosolic ligand-inducible glucocorticoid receptors. Inactive glucocorticoid receptors are associated with (co)chaperones, including Hsp90, which dissociate after their ligation, followed by nuclear translocation of these receptors and regulation of gene transcription [21]. The glucocorticoid receptor has been described as part of a T cell receptor-linked multiprotein complex containing Hsp90 and the nonreceptor tyrosin kinases Lck and Fyn, which is essential for T cell receptor-dependent Lck/Fyn activation. It has been previously shown that either treatment with dexamethasone or knocking down Hsp90 by Hsp90siRNA induces dissociation of this protein complex, resulting in abrogated T cell receptor signaling as a consequence of impaired Lck/Fyn activation [22]. Similar to this and other previous studies [4,13,22], we could show in the current experiments that pharmacological blockade of Hsp90 was associated with inhibition of Lck activation in anti-CD3 antibody-stimulated PBMCs, a mechanism that could further account for the observed immunosuppressive effects of this treatment on T cells.

Considering that inhibition of both NF-kB function and proximal T cell receptor signaling by corticosteroids can be mimicked using Hsp90 inhibitors and that a novel generation of Hsp90 blockers with good tolerability has been reported in the field of cancer treatment [4,13,22-25], it remains to be clarified in the future whether this class of drugs can potentially represent an effective alternative for corticosteroid therapy with a better side effect profile in patients with autoimmune diseases.

Together, our results further support the potential use of Hsp90 inhibitors in patients with autoimmune diseases where inappropriate activation of proinflammatory Th1 and Th17 subpopulations frequently occurs.

## Competing interests

The authors declare that they have no competing interests.

## Authors’ contributions

ST conceived the study, designed experiments, performed research, analyzed data, and wrote the manuscript. DZ analyzed data and contributed to composition of the paper. MK helped conceive of the study, design experiments, and write the manuscript. All authors read and approved the final manuscript.

## References

[B1] Taipale M Jarosz DF Lindquist S HSP90 at the hub of protein homeostasis: emerging mechanistic insights Nat Rev Mol Cell Biol 2010 11 515 528 10.1038/nrm2918 20531426

[B2] Srivastava P Roles of heat-shock proteins in innate and adaptive immunity Nat Rev Immunol 2002 2 185 194 10.1038/nri749 11913069

[B3] Ulivieri C Baldari CT T-cell-based immunotherapy of autoimmune diseases Expert Rev Vaccines 2013 12 297 310 10.1586/erv.12.146 23496669

[B4] Bae J Munshi A Li C Samur M Prabhala R Mitsiades C Anderson KC Munshi NC Heat shock protein 90 is critical for regulation of phenotype and functional activity of human T lymphocytes and NK cells J Immunol 2013 190 1360 1371 10.4049/jimmunol.1200593 23293352 PMC3819808

[B5] Wang C Wu L Bulek K Martin BN Zepp JA Kang Z Liu C Herjan T Misra S Carman JA Gao J Dongre A Han S Bunting KD Ko JS Xiao H Kuchroo VK Ouyang W Li X The psoriasis-associated D10N variant of the adaptor Act1 with impaired regulation by the molecular chaperone hsp90 Nat Immunol 2013 14 72 81 23202271 10.1038/ni.2479 PMC3522792

[B6] Dello Russo C Polak PE Mercado PR Spagnolo A Sharp A Murphy P Kamal A Burrows FJ Fritz LC Feinstein DL The heat-shock protein 90 inhibitor 17-allylamino-17-demethoxygeldanamycin suppresses glial inflammatory responses and ameliorates experimental autoimmune encephalomyelitis J Neurochem 2006 99 1351 1362 10.1111/j.1471-4159.2006.04221.x 17064348

[B7] Rice JW Veal JM Fadden RP Barabasz AF Partridge JM Barta TE Dubois LG Huang KH Mabbett SR Silinski MA Steed PM Hall SE Small molecule inhibitors of Hsp90 potently affect inflammatory disease pathways and exhibit activity in models of rheumatoid arthritis Arthritis Rheum 2008 58 3765 3775 10.1002/art.24047 19035474

[B8] Yun TJ Harning EK Giza K Rabah D Li P Arndt JW Luchetti D Biamonte MA Shi J Lundgren K Manning A Kehry MR EC144, a synthetic inhibitor of heat shock protein 90, blocks innate and adaptive immune responses in models of inflammation and autoimmunity J Immunol 2011 186 563 575 10.4049/jimmunol.1000222 21131419

[B9] Han JM Kwon NH Lee JY Jeong SJ Jung HJ Kim HR Li Z Kim S Identification of gp96 as a novel target for treatment of autoimmune disease in mice PLoS One 2010 5 e9792 10.1371/journal.pone.0009792 20352117 PMC2843739

[B10] Shimp SK 3rd Chafin CB Regna NL Hammond SE Read MA Caudell DL Rylander M Reilly CM Heat shock protein 90 inhibition by 17-DMAG lessens disease in the MRL/lpr mouse model of systemic lupus erythematosus Cell Mol Immunol 2012 9 255 266 10.1038/cmi.2012.5 22543833 PMC4012849

[B11] Kasperkiewicz M Müller R Manz R Magens M Hammers CM Somlai C Westermann J Schmidt E Zillikens D Ludwig RJ Orosz A Heat-shock protein 90 inhibition in autoimmunity to type VII collagen: evidence that nonmalignant plasma cells are not therapeutic targets Blood 2011 117 6135 6142 10.1182/blood-2010-10-314609 21490339

[B12] Tukaj S Kleszczyński K Vafia K Groth S Meyersburg D Trzonkowski P Ludwig RJ Zillikens D Schmidt E Fischer TW Kasperkiewicz M Aberrant expression and secretion of heat shock protein 90 in patients with bullous pemphigoid PLoS One 2013 8 e70496 10.1371/journal.pone.0070496 23936217 PMC3728143

[B13] Yorgin PD Hartson SD Fellah AM Scroggins BT Huang W Katsanis E Couchman JM Matts RL Whitesell L Effects of geldanamycin, a heat-shock protein 90-binding agent, on T cell function and T cell nonreceptor protein tyrosine kinases J Immunol 2000 164 2915 2923 10706677 10.4049/jimmunol.164.6.2915

[B14] De Zoeten EF Wang L Butler K Beier UH Akimova T Sai H Bradner JE Mazitschek R Kozikowski AP Matthias P Hancock WW Histone deacetylase 6 and heat shock protein 90 control the functions of Foxp3(+) T-regulatory cells Mol Cell Biol 2011 31 2066 2078 10.1128/MCB.05155-11 21444725 PMC3133361

[B15] Collins CB Aherne CM Yeckes A Pound K Eltzschig HK Jedlicka P De Zoeten EF Inhibition of N-terminal ATPase on HSP90 attenuates colitis through enhanced Treg function Mucosal Immunol 2013 6 960 971 10.1038/mi.2012.134 23321985 PMC3748235

[B16] Salminen A Paimela T Suuronen T Kaarniranta K Innate immunity meets with cellular stress at the IKK complex: regulation of the IKK complex by HSP70 and HSP90 Immunol Lett 2008 117 9 15 10.1016/j.imlet.2007.12.017 18282612

[B17] Oh H Ghosh S NF-κB: roles and regulation in different CD4(+) T-cell subsets Immunol Rev 2013 252 41 51 10.1111/imr.12033 23405894 PMC3576882

[B18] Dakappagari N Neely L Tangri S Lundgren K Hipolito L Estrellado A Burrows F Zhang H An investigation into the potential use of serum Hsp70 as a novel tumour biomarker for Hsp90 inhibitors Biomarkers 2010 5 31 38 19747088 10.3109/13547500903261347

[B19] Stocki P Dickinson AM The immunosuppressive activity of heat shock protein 70 Autoimmune Dis 2012 2012 617213 23326648 10.1155/2012/617213 PMC3533589

[B20] De Jong PR Schadenberg AW Jansen NJ Prakken BJ Hsp70 and cardiac surgery: molecular chaperone and inflammatory regulator with compartmentalized effects Cell Stress Chaperones 2009 14 117 131 10.1007/s12192-008-0066-9 18668350 PMC2727984

[B21] Pratt WB Galigniana MD Morishima Y Murphy PJ Role of molecular chaperones in steroid receptor action Essays Biochem 2004 40 41 58 15242338 10.1042/bse0400041

[B22] Löwenberg M Verhaar AP Bilderbeek J Marle J Buttgereit F Peppelenbosch MP Van Deventer SJ Hommes DW Glucocorticoids cause rapid dissociation of a T-cell-receptor-associated protein complex containing LCK and FYN EMBO Rep 2006 7 1023 1029 10.1038/sj.embor.7400775 16888650 PMC1618362

[B23] Celec P Nuclear factor kappa B–molecular biomedicine: the next generation Biomed Pharmacother 2004 58 365 371 10.1016/j.biopha.2003.12.015 15271418

[B24] Orosz A Szabo A Szeman G Janaky T Somlai C Penke B Bodor A Perczel A Novel nontoxic heat shock protein 90 inhibitors having selective antiproliferative effect Int J Biochem Cell Biol 2006 38 1352 1362 10.1016/j.biocel.2006.01.015 16540363

[B25] Garcia-Carbonero R Carnero A Paz-Ares L Inhibition of HSP90 molecular chaperones: moving into the clinic Lancet Oncol 2013 14 e358 e369 10.1016/S1470-2045(13)70169-4 23896275

